# Tris(2,2′-bipyridine)­nickel(II) hexa­molybdate

**DOI:** 10.1107/S1600536810032058

**Published:** 2010-08-18

**Authors:** Liming Fan, Peihai Wei, Shuming Pang, Xiutang Zhang

**Affiliations:** a Advanced Material Institute of Research, Department of Chemistry and Chemical Engineering, Qilu Normal University, Jinan 250013, People’s Republic of China; b College of Chemistry and Chemical Engineering, Liaocheng University, Liaocheng 252059, People’s Republic of China

## Abstract

The asymmetric unit of the title compound, [Ni(C_10_H_8_N_2_)_3_][Mo_6_O_19_], consists of one complex [(Ni(C_10_H_8_N_2_)_3_]^2+^ cation and one Lindqvist-type [Mo_6_O_19_]^2−^ polyanion. The Ni^2+^ ion is in a distorted octa­hedral coordination by six N atoms from three chelating 2,2′-bipyridine ligands. The Lindqvist-type anion exhibits the characteristic Mo—O bond-length distribution, with the shortest bonds being the Mo—O(terminal) bonds [mean = 1.679 (2) Å] and the longest being those to the central O atom [mean = 2.318 (7) Å]. A number of C—H⋯O inter­actions contribute to the crystal packing.

## Related literature

For background to polyoxidometalates, see: Pope & Müller (1991 ▶). For polyoxidometalates modified with amines, see: Zhang *et al.* (2009**a* ▶,b* ▶). For other Lindqvist-type [Mo_6_O_19_]^2−^ anions, see: Che *et al.* (1979 ▶); Pope (1983 ▶).
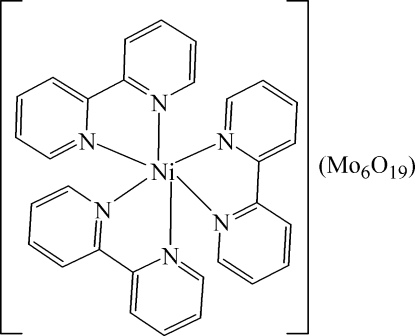


## Experimental

### 

#### Crystal data

[Ni(C_10_H_8_N_2_)_3_][Mo_6_O_19_]
*M* *_r_* = 1406.90
Monoclinic, 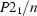

*a* = 12.3549 (7) Å
*b* = 18.9866 (10) Å
*c* = 17.1974 (9) Å
β = 101.114 (1)°
*V* = 3958.5 (4) Å^3^
*Z* = 4
Mo *K*α radiation
μ = 2.39 mm^−1^
*T* = 296 K
0.12 × 0.10 × 0.08 mm

#### Data collection

Bruker APEXII CCD diffractometer
Absorption correction: multi-scan (*SADABS*; Bruker, 2001 ▶) *T*_min_ = 0.762, *T*_max_ = 0.832
27627 measured reflections
6968 independent reflections
5819 reflections with *I* > 2σ(*I*)
*R*_int_ = 0.027

#### Refinement

*R*[*F* ^2^ > 2σ(*F* ^2^)] = 0.023
*wR*(*F* ^2^) = 0.072
*S* = 1.00
6968 reflections
559 parameters
H-atom parameters constrained
Δρ_max_ = 0.47 e Å^−3^
Δρ_min_ = −0.54 e Å^−3^

### 

Data collection: *APEX2* (Bruker, 2004 ▶); cell refinement: *SAINT-Plus* (Bruker, 2001 ▶); data reduction: *SAINT-Plus*; program(s) used to solve structure: *SHELXS97* (Sheldrick, 2008 ▶); program(s) used to refine structure: *SHELXL97* (Sheldrick, 2008 ▶); molecular graphics: *SHELXTL* (Sheldrick, 2008 ▶); software used to prepare material for publication: *SHELXTL*.

## Supplementary Material

Crystal structure: contains datablocks global, I. DOI: 10.1107/S1600536810032058/wm2381sup1.cif

Structure factors: contains datablocks I. DOI: 10.1107/S1600536810032058/wm2381Isup2.hkl

Additional supplementary materials:  crystallographic information; 3D view; checkCIF report

## Figures and Tables

**Table 1:** Selected bond lengths (Å)

N4—Ni1	2.066 (3)
N3—Ni1	2.078 (3)
N2—Ni1	2.082 (3)
N6—Ni1	2.095 (3)
N1—Ni1	2.101 (3)
N5—Ni1	2.105 (3)

**Table 2:** Hydrogen-bond geometry (Å, °)

*D*—H⋯*A*	*D*—H	H⋯*A*	*D*⋯*A*	*D*—H⋯*A*
C29—H29⋯O11^i^	0.93	2.48	3.293 (5)	146
C2—H2⋯O1^ii^	0.93	2.66	3.381 (5)	134
C14—H14⋯O5^ii^	0.93	2.67	3.386 (4)	135
C14—H14⋯O11^ii^	0.93	2.75	3.396 (4)	127
C8—H8⋯O2^iii^	0.93	2.66	3.228 (5)	120
C7—H7⋯O2^iii^	0.93	2.53	3.169 (4)	126
C12—H12⋯O16^iv^	0.93	2.54	3.241 (4)	132
C9—H9⋯O15^v^	0.93	2.36	3.165 (4)	144
C21—H21⋯O3^vi^	0.93	2.54	3.163 (5)	124

Symmetry codes: (i) 
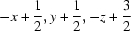
; (ii) 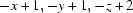; (iii) 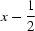, 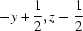; (iv) 
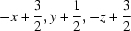
; (v) 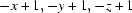; (vi) 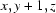.

**Fig. 1.**
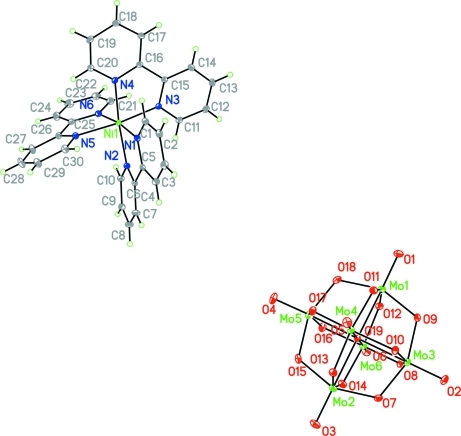
The cation and anion of the title compound with the atom-labelling scheme. Displacement ellipsoids are drawn at the 30% probability level; H atoms are given as spheres of arbitrary radius.

**Fig. 2.**
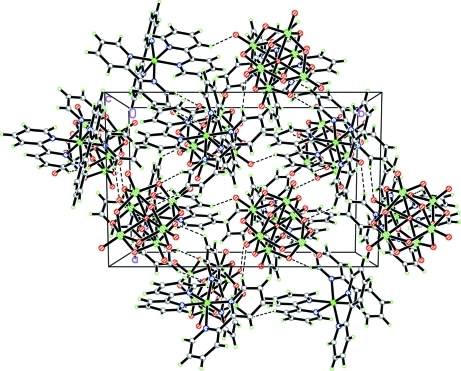
The crystal packing of the title compound, displayed with C—H···O hydrogen bonds as dashed lines.

## supplementary crystallographic information

### Comment

The design and synthesis of polyoxidometalates has attracted continuous research interest not only because of their appealing structural and topological novelties, but also due to their interesting optical, electronic, magnetic, and catalytic properties, as well as their potential medical applications (Pope & Müller, 1991). In our group, organic amines, such as 3-(2-pyridyl)pyrazole and pyrazine, are used to effectively modify polyoxidomolybdates under hydrothermal conditions (Zhang *et al.*, 2009**a*,b*). Here, we describe the synthesis and structural characterization of the title compound, [(Ni(C_10_H_8_N_2_)_3_][Mo_6_O_19_].

The title compound consists of one complex [(Ni(C_10_H_8_N_2_)_3_]^2+^ cation and one Lindqvist-type [Mo_6_O_19_]^2-^ polyanion, as shown in Figure 1. The nickel(II) ion is in a distorted octahedral coordination by six N atoms from three chelating 2,2'-bipyridine ligands. The Ni—N bond lengths are in the normal range of 2.066 (3)—2.101 (3) Å and are also in agreement with previous studies (Zhang *et al.*, 2009*a*). The rms deviations of the three bipyridine groups from planarity are 0.0779, 0.0178, and 0.0806 Å, respectively.

The hexamolybdate anion is of the Lindqvist-type (Pope, 1983; Che *et al.*, 1979) and typically consists of six molybdenum atoms arranged octahedrally around a central oxygen atom. Each molybdenum is then bonded peripherally to neighboring molybdenum atoms through oxygen bridges. One terminal oxygen atom is attached to each molybdenum atom. Alternatively, the structure can be visualized as formed from six MoO_6_ octahedra that have condensed so that they all share a common vertex.

Multipoint C—H···O interactions between the hydrogen atoms from the organic amines and the terminal oxygen atoms of the anion make a contribution to stabilize the packing of the crystal, as shown in Figure 2 and Table 2.

### Experimental

A mixture of sodium molybdate dihydrate (0.04 mmol, 0.10 g), 2,2'-bipyridine (0.32 mmol, 0.05 g), nickel dichloride hexahydrate (0.21 mmol, 0.05 g), and 2-(3'-carboxy-phenoxy)benzoic acid (0.20 mmoL, 0.05 g), and 14 ml H_2_O was sealed in a 25 ml Teflon-lined stainless steel autoclave at 433 K for three days. Green crystals suitable for the X-ray experiment were obtained. Anal. Calc. for C_30_H_24_Mo_6_N_6_NiO_19_: C 25.59, H 1.71, N 5.97%; Found: C 25.48, H 1.65, N 5.88%.

### Refinement

All hydrogen atoms bound to carbon were refined using a riding model with distance C—H = 0.93 Å and *U*_iso_(H) = 1.2*U*_eq_(C).

### Crystal data

**Table:**

[Ni(C_10_H_8_N_2_)_3_][Mo_6_O_19_]	*F*(000) = 2712
*M**_r_* = 1406.90	*D*_x_ = 2.361 Mg m^−^^3^
Monoclinic, *P*2_1_/*n*	Mo *K*α radiation, λ = 0.71073 Å
Hall symbol: -P 2yn	Cell parameters from 9988 reflections
*a* = 12.3549 (7) Å	θ = 2.3–26.7°
*b* = 18.9866 (10) Å	µ = 2.39 mm^−^^1^
*c* = 17.1974 (9) Å	*T* = 296 K
β = 101.114 (1)°	Block, green
*V* = 3958.5 (4) Å^3^	0.12 × 0.10 × 0.08 mm
*Z* = 4	

### Data collection

**Table:**

Bruker APEXII CCD diffractometer	6968 independent reflections
Radiation source: fine-focus sealed tube	5819 reflections with *I* > 2σ(*I*)
graphite	*R*_int_ = 0.027
phi and ω scans	θ_max_ = 25.0°, θ_min_ = 2.2°
Absorption correction: multi-scan (*SADABS*; Bruker, 2001)	*h* = −14→14
*T*_min_ = 0.762, *T*_max_ = 0.832	*k* = −22→22
27627 measured reflections	*l* = −20→20

### Refinement

**Table:**

Refinement on *F*^2^	Primary atom site location: structure-invariant direct methods
Least-squares matrix: full	Secondary atom site location: difference Fourier map
*R*[*F*^2^ > 2σ(*F*^2^)] = 0.023	Hydrogen site location: inferred from neighbouring sites
*wR*(*F*^2^) = 0.072	H-atom parameters constrained
*S* = 1.00	*w* = 1/[σ^2^(*F*_o_^2^) + (0.047*P*)^2^ + 0.0562*P*] where *P* = (*F*_o_^2^ + 2*F*_c_^2^)/3
6968 reflections	(Δ/σ)_max_ = 0.009
559 parameters	Δρ_max_ = 0.47 e Å^−^^3^
0 restraints	Δρ_min_ = −0.54 e Å^−^^3^

### Special details

**Table:**

Geometry. All e.s.d.'s (except the e.s.d. in the dihedral angle between two l.s. planes) are estimated using the full covariance matrix. The cell e.s.d.'s are taken into account individually in the estimation of e.s.d.'s in distances, angles and torsion angles; correlations between e.s.d.'s in cell parameters are only used when they are defined by crystal symmetry. An approximate (isotropic) treatment of cell e.s.d.'s is used for estimating e.s.d.'s involving l.s. planes.
Refinement. Refinement of *F*^2^ against ALL reflections. The weighted *R*-factor *wR* and goodness of fit *S* are based on *F*^2^, conventional *R*-factors *R* are based on *F*, with *F* set to zero for negative *F*^2^. The threshold expression of *F*^2^ > σ(*F*^2^) is used only for calculating *R*-factors(gt) *etc*. and is not relevant to the choice of reflections for refinement. *R*-factors based on *F*^2^ are statistically about twice as large as those based on *F*, and *R*- factors based on ALL data will be even larger.

### Fractional atomic coordinates and isotropic or equivalent isotropic displacement parameters (Å^2^)

**Table:**

	*x*	*y*	*z*	*U*_iso_*/*U*_eq_	
C1	0.2047 (3)	0.7484 (2)	0.7553 (2)	0.0372 (8)	
H1	0.1832	0.7861	0.7834	0.045*	
C2	0.1962 (3)	0.6817 (2)	0.7838 (2)	0.0427 (9)	
H2	0.1696	0.6743	0.8302	0.051*	
C3	0.2280 (3)	0.6259 (2)	0.7419 (2)	0.0477 (10)	
H3	0.2229	0.5799	0.7596	0.057*	
C4	0.2672 (3)	0.63901 (19)	0.6741 (2)	0.0437 (9)	
H4	0.2883	0.6019	0.6450	0.052*	
C5	0.2752 (3)	0.70811 (17)	0.6490 (2)	0.0313 (8)	
C6	0.3212 (3)	0.72633 (18)	0.5787 (2)	0.0342 (8)	
C7	0.3484 (3)	0.6771 (2)	0.5263 (2)	0.0471 (10)	
H7	0.3358	0.6295	0.5334	0.057*	
C8	0.3943 (4)	0.6991 (2)	0.4636 (2)	0.0530 (11)	
H8	0.4127	0.6666	0.4279	0.064*	
C9	0.4126 (3)	0.7691 (2)	0.4546 (2)	0.0469 (10)	
H9	0.4441	0.7850	0.4129	0.056*	
C10	0.3838 (3)	0.8157 (2)	0.5080 (2)	0.0405 (9)	
H10	0.3962	0.8635	0.5013	0.049*	
C11	0.5056 (3)	0.85816 (19)	0.7315 (2)	0.0409 (9)	
H11	0.5233	0.8418	0.6845	0.049*	
C12	0.5877 (3)	0.8614 (2)	0.7979 (3)	0.0469 (10)	
H12	0.6594	0.8479	0.7956	0.056*	
C13	0.5618 (3)	0.8847 (2)	0.8674 (3)	0.0478 (10)	
H13	0.6158	0.8873	0.9132	0.057*	
C14	0.4549 (3)	0.90433 (19)	0.8687 (2)	0.0427 (9)	
H14	0.4356	0.9193	0.9157	0.051*	
C15	0.3769 (3)	0.90155 (17)	0.7999 (2)	0.0310 (7)	
C16	0.2599 (3)	0.92287 (16)	0.7947 (2)	0.0301 (7)	
C17	0.2196 (3)	0.94944 (19)	0.8589 (2)	0.0411 (9)	
H17	0.2662	0.9555	0.9078	0.049*	
C18	0.1088 (3)	0.9667 (2)	0.8489 (2)	0.0447 (9)	
H18	0.0802	0.9844	0.8912	0.054*	
C19	0.0415 (3)	0.9576 (2)	0.7760 (2)	0.0459 (10)	
H19	−0.0329	0.9693	0.7678	0.055*	
C20	0.0872 (3)	0.93069 (19)	0.7155 (2)	0.0407 (9)	
H20	0.0418	0.9243	0.6662	0.049*	
C21	0.3602 (3)	1.00319 (19)	0.5998 (2)	0.0455 (9)	
H21	0.4128	0.9962	0.6457	0.055*	
C22	0.3655 (4)	1.0633 (2)	0.5560 (3)	0.0539 (11)	
H22	0.4210	1.0962	0.5719	0.065*	
C23	0.2880 (4)	1.0738 (2)	0.4886 (3)	0.0653 (13)	
H23	0.2898	1.1140	0.4581	0.078*	
C24	0.2077 (4)	1.0237 (2)	0.4670 (3)	0.0570 (11)	
H24	0.1540	1.0303	0.4216	0.068*	
C25	0.2060 (3)	0.96389 (18)	0.5121 (2)	0.0360 (8)	
C26	0.1217 (3)	0.90776 (18)	0.4935 (2)	0.0349 (8)	
C27	0.0483 (3)	0.9037 (2)	0.4220 (2)	0.0529 (11)	
H27	0.0494	0.9374	0.3828	0.064*	
C28	−0.0262 (4)	0.8494 (3)	0.4097 (2)	0.0641 (13)	
H28	−0.0764	0.8461	0.3620	0.077*	
C29	−0.0265 (3)	0.8002 (2)	0.4676 (2)	0.0554 (11)	
H29	−0.0772	0.7634	0.4602	0.066*	
C30	0.0501 (3)	0.8061 (2)	0.5371 (2)	0.0439 (9)	
H30	0.0504	0.7725	0.5765	0.053*	
Mo1	0.78107 (3)	0.201308 (17)	0.957341 (19)	0.03791 (9)	
Mo2	0.66433 (3)	0.063859 (18)	0.733433 (19)	0.03975 (10)	
Mo3	0.82030 (2)	0.038531 (16)	0.907938 (19)	0.03494 (9)	
Mo4	0.56817 (2)	0.097421 (17)	0.894435 (19)	0.03583 (9)	
Mo5	0.62406 (3)	0.227585 (18)	0.78320 (2)	0.04239 (10)	
Mo6	0.87528 (2)	0.167533 (17)	0.795574 (19)	0.03537 (9)	
N1	0.2427 (2)	0.76195 (14)	0.68892 (16)	0.0305 (6)	
N2	0.3387 (2)	0.79591 (15)	0.56930 (16)	0.0328 (7)	
N3	0.4013 (2)	0.87750 (14)	0.73126 (16)	0.0320 (6)	
N4	0.1938 (2)	0.91327 (14)	0.72410 (17)	0.0329 (6)	
N5	0.1243 (2)	0.85861 (15)	0.55014 (16)	0.0336 (6)	
N6	0.2817 (2)	0.95434 (14)	0.57853 (17)	0.0336 (7)	
Ni1	0.26519 (3)	0.86154 (2)	0.64110 (2)	0.02895 (11)	
O1	0.8233 (2)	0.25281 (15)	1.03673 (16)	0.0553 (7)	
O2	0.8935 (2)	−0.02945 (14)	0.95332 (18)	0.0530 (7)	
O3	0.6193 (2)	0.01285 (17)	0.65399 (17)	0.0612 (8)	
O4	0.5487 (2)	0.29421 (16)	0.73646 (19)	0.0661 (9)	
O5	0.4599 (2)	0.07256 (15)	0.93400 (16)	0.0505 (7)	
O6	0.9841 (2)	0.19293 (15)	0.75756 (17)	0.0508 (7)	
O7	0.7549 (2)	0.00033 (12)	0.80675 (15)	0.0426 (6)	
O8	0.92547 (19)	0.08525 (12)	0.85333 (15)	0.0382 (6)	
O9	0.85473 (18)	0.11392 (13)	0.98427 (14)	0.0385 (6)	
O10	0.68230 (19)	0.02940 (12)	0.93946 (14)	0.0380 (6)	
O11	0.6499 (2)	0.16021 (13)	0.97714 (14)	0.0406 (6)	
O12	0.89135 (19)	0.21893 (12)	0.89103 (15)	0.0388 (6)	
O13	0.55100 (19)	0.04720 (13)	0.79866 (15)	0.0400 (6)	
O14	0.7956 (2)	0.10435 (14)	0.71345 (14)	0.0444 (6)	
O15	0.5892 (2)	0.15067 (14)	0.70706 (14)	0.0457 (6)	
O16	0.7612 (2)	0.23554 (13)	0.75091 (15)	0.0452 (6)	
O17	0.51973 (19)	0.18101 (13)	0.83857 (15)	0.0428 (6)	
O18	0.6892 (2)	0.26559 (12)	0.88422 (16)	0.0466 (6)	
O19	0.72228 (16)	0.13273 (10)	0.84515 (12)	0.0287 (5)	

### Atomic displacement parameters (Å^2^)

**Table:**

	*U*^11^	*U*^22^	*U*^33^	*U*^12^	*U*^13^	*U*^23^
C1	0.0354 (19)	0.045 (2)	0.031 (2)	−0.0048 (16)	0.0068 (16)	−0.0007 (16)
C2	0.043 (2)	0.049 (2)	0.035 (2)	−0.0051 (18)	0.0053 (17)	0.0113 (18)
C3	0.052 (2)	0.038 (2)	0.052 (3)	−0.0061 (18)	0.007 (2)	0.0122 (19)
C4	0.052 (2)	0.031 (2)	0.047 (2)	−0.0004 (17)	0.0077 (19)	−0.0009 (17)
C5	0.0310 (18)	0.0308 (18)	0.0292 (18)	0.0004 (14)	−0.0018 (14)	−0.0009 (15)
C6	0.0374 (19)	0.034 (2)	0.0293 (19)	0.0038 (15)	0.0024 (15)	0.0001 (15)
C7	0.070 (3)	0.033 (2)	0.041 (2)	0.0031 (19)	0.017 (2)	−0.0066 (17)
C8	0.072 (3)	0.049 (3)	0.042 (2)	0.010 (2)	0.019 (2)	−0.0105 (19)
C9	0.053 (2)	0.059 (3)	0.031 (2)	0.012 (2)	0.0124 (18)	0.0000 (18)
C10	0.049 (2)	0.041 (2)	0.035 (2)	0.0021 (17)	0.0152 (18)	0.0047 (16)
C11	0.037 (2)	0.039 (2)	0.049 (2)	0.0037 (16)	0.0136 (18)	−0.0044 (17)
C12	0.0312 (19)	0.042 (2)	0.065 (3)	0.0029 (17)	0.0023 (19)	−0.005 (2)
C13	0.039 (2)	0.043 (2)	0.054 (3)	0.0010 (18)	−0.0102 (19)	−0.0046 (19)
C14	0.048 (2)	0.043 (2)	0.035 (2)	−0.0041 (18)	0.0024 (17)	−0.0071 (17)
C15	0.0348 (18)	0.0252 (17)	0.0335 (19)	−0.0018 (14)	0.0080 (15)	0.0007 (14)
C16	0.0398 (19)	0.0212 (16)	0.0306 (19)	−0.0001 (14)	0.0101 (15)	0.0036 (14)
C17	0.049 (2)	0.040 (2)	0.035 (2)	0.0059 (17)	0.0096 (17)	−0.0043 (16)
C18	0.048 (2)	0.047 (2)	0.043 (2)	0.0073 (18)	0.0213 (19)	−0.0042 (18)
C19	0.036 (2)	0.048 (2)	0.056 (3)	0.0133 (18)	0.0144 (19)	0.0000 (19)
C20	0.039 (2)	0.045 (2)	0.036 (2)	0.0075 (17)	0.0029 (17)	−0.0038 (17)
C21	0.045 (2)	0.041 (2)	0.051 (2)	−0.0072 (18)	0.0105 (19)	−0.0045 (19)
C22	0.065 (3)	0.034 (2)	0.067 (3)	−0.014 (2)	0.022 (2)	−0.005 (2)
C23	0.094 (4)	0.037 (2)	0.070 (3)	−0.006 (2)	0.027 (3)	0.014 (2)
C24	0.070 (3)	0.045 (2)	0.052 (3)	0.002 (2)	0.002 (2)	0.016 (2)
C25	0.043 (2)	0.0325 (19)	0.034 (2)	0.0056 (16)	0.0113 (16)	0.0015 (15)
C26	0.0335 (19)	0.040 (2)	0.0321 (19)	0.0030 (15)	0.0080 (15)	0.0051 (16)
C27	0.048 (2)	0.072 (3)	0.037 (2)	−0.009 (2)	0.0019 (19)	0.012 (2)
C28	0.048 (3)	0.105 (4)	0.035 (2)	−0.019 (3)	−0.0030 (19)	0.001 (2)
C29	0.042 (2)	0.075 (3)	0.048 (3)	−0.023 (2)	0.007 (2)	−0.006 (2)
C30	0.038 (2)	0.051 (2)	0.044 (2)	−0.0113 (18)	0.0117 (18)	0.0042 (18)
Mo1	0.03947 (19)	0.03603 (18)	0.03897 (19)	−0.00078 (14)	0.00938 (15)	−0.00888 (14)
Mo2	0.03436 (18)	0.0506 (2)	0.03378 (19)	−0.00618 (14)	0.00537 (14)	−0.00796 (15)
Mo3	0.03277 (17)	0.03014 (17)	0.04154 (19)	0.00591 (13)	0.00619 (14)	0.00551 (13)
Mo4	0.02880 (17)	0.04349 (19)	0.03698 (19)	0.00007 (13)	0.01082 (13)	0.00503 (14)
Mo5	0.03764 (19)	0.0421 (2)	0.0484 (2)	0.01227 (14)	0.01073 (15)	0.01602 (15)
Mo6	0.03013 (17)	0.03729 (18)	0.04096 (19)	−0.00224 (13)	0.01251 (14)	0.00231 (14)
N1	0.0344 (15)	0.0301 (15)	0.0261 (15)	−0.0028 (12)	0.0033 (12)	0.0019 (12)
N2	0.0369 (16)	0.0336 (16)	0.0287 (16)	0.0019 (13)	0.0080 (13)	−0.0001 (12)
N3	0.0312 (15)	0.0300 (15)	0.0344 (16)	0.0018 (12)	0.0054 (13)	−0.0015 (12)
N4	0.0319 (15)	0.0318 (15)	0.0350 (17)	0.0048 (12)	0.0064 (13)	0.0019 (12)
N5	0.0311 (15)	0.0364 (16)	0.0335 (16)	−0.0016 (13)	0.0069 (13)	0.0005 (13)
N6	0.0352 (16)	0.0285 (15)	0.0378 (17)	−0.0021 (12)	0.0089 (13)	−0.0007 (13)
Ni1	0.0297 (2)	0.0287 (2)	0.0287 (2)	0.00063 (18)	0.00644 (18)	−0.00019 (18)
O1	0.0588 (18)	0.0545 (17)	0.0537 (18)	−0.0036 (14)	0.0139 (15)	−0.0208 (14)
O2	0.0491 (16)	0.0368 (14)	0.071 (2)	0.0091 (12)	0.0052 (14)	0.0127 (14)
O3	0.0499 (17)	0.083 (2)	0.0496 (18)	−0.0156 (16)	0.0074 (14)	−0.0250 (16)
O4	0.0562 (18)	0.0578 (19)	0.083 (2)	0.0200 (15)	0.0104 (17)	0.0318 (17)
O5	0.0364 (15)	0.0683 (19)	0.0500 (17)	−0.0068 (13)	0.0166 (13)	0.0075 (14)
O6	0.0387 (15)	0.0616 (18)	0.0562 (18)	−0.0080 (13)	0.0190 (13)	0.0004 (14)
O7	0.0456 (15)	0.0323 (13)	0.0498 (16)	−0.0011 (11)	0.0088 (12)	−0.0086 (12)
O8	0.0291 (12)	0.0359 (13)	0.0509 (16)	0.0035 (10)	0.0107 (11)	−0.0012 (12)
O9	0.0338 (13)	0.0443 (14)	0.0343 (14)	0.0037 (11)	−0.0011 (11)	0.0012 (11)
O10	0.0367 (13)	0.0373 (14)	0.0397 (14)	−0.0002 (11)	0.0069 (11)	0.0099 (11)
O11	0.0406 (14)	0.0464 (15)	0.0374 (14)	0.0032 (11)	0.0138 (12)	−0.0033 (12)
O12	0.0347 (13)	0.0365 (13)	0.0460 (15)	−0.0058 (11)	0.0095 (11)	−0.0041 (11)
O13	0.0340 (13)	0.0445 (15)	0.0409 (15)	−0.0064 (11)	0.0055 (11)	−0.0028 (12)
O14	0.0385 (14)	0.0629 (17)	0.0352 (14)	−0.0063 (13)	0.0155 (12)	−0.0075 (13)
O15	0.0370 (14)	0.0622 (17)	0.0351 (14)	0.0028 (12)	0.0001 (11)	0.0132 (13)
O16	0.0437 (15)	0.0428 (15)	0.0516 (16)	−0.0004 (12)	0.0153 (13)	0.0189 (12)
O17	0.0329 (13)	0.0500 (15)	0.0471 (15)	0.0098 (12)	0.0117 (12)	0.0082 (12)
O18	0.0501 (16)	0.0320 (13)	0.0602 (18)	0.0073 (12)	0.0165 (13)	−0.0011 (12)
O19	0.0266 (12)	0.0287 (12)	0.0313 (12)	0.0019 (9)	0.0069 (10)	0.0021 (10)

### Geometric parameters (Å, °)

**Table:**

C1—N1	1.341 (4)	C26—N5	1.344 (4)
C1—C2	1.369 (5)	C26—C27	1.382 (5)
C1—H1	0.9300	C27—C28	1.371 (6)
C2—C3	1.381 (5)	C27—H27	0.9300
C2—H2	0.9300	C28—C29	1.367 (6)
C3—C4	1.369 (5)	C28—H28	0.9300
C3—H3	0.9300	C29—C30	1.379 (5)
C4—C5	1.391 (5)	C29—H29	0.9300
C4—H4	0.9300	C30—N5	1.343 (4)
C5—N1	1.336 (4)	C30—H30	0.9300
C5—C6	1.472 (5)	Mo1—O1	1.679 (3)
C6—N2	1.353 (4)	Mo1—O11	1.888 (2)
C6—C7	1.383 (5)	Mo1—O9	1.906 (2)
C7—C8	1.377 (6)	Mo1—O18	1.952 (3)
C7—H7	0.9300	Mo1—O12	1.967 (2)
C8—C9	1.361 (6)	Mo1—O19	2.325 (2)
C8—H8	0.9300	Mo2—O3	1.680 (3)
C9—C10	1.371 (5)	Mo2—O14	1.885 (2)
C9—H9	0.9300	Mo2—O15	1.903 (3)
C10—N2	1.338 (4)	Mo2—O7	1.937 (2)
C10—H10	0.9300	Mo2—O13	1.981 (2)
C11—N3	1.339 (4)	Mo2—O19	2.320 (2)
C11—C12	1.374 (5)	Mo3—O2	1.680 (2)
C11—H11	0.9300	Mo3—O10	1.894 (2)
C12—C13	1.370 (6)	Mo3—O7	1.914 (2)
C12—H12	0.9300	Mo3—O9	1.933 (2)
C13—C14	1.377 (5)	Mo3—O8	1.956 (2)
C13—H13	0.9300	Mo3—O19	2.308 (2)
C14—C15	1.375 (5)	Mo4—O5	1.681 (2)
C14—H14	0.9300	Mo4—O13	1.879 (2)
C15—N3	1.353 (4)	Mo4—O17	1.892 (2)
C15—C16	1.487 (5)	Mo4—O10	1.958 (2)
C16—N4	1.339 (4)	Mo4—O11	1.977 (2)
C16—C17	1.390 (5)	Mo4—O19	2.327 (2)
C17—C18	1.385 (5)	Mo5—O4	1.680 (3)
C17—H17	0.9300	Mo5—O16	1.888 (2)
C18—C19	1.376 (5)	Mo5—O18	1.910 (3)
C18—H18	0.9300	Mo5—O15	1.953 (3)
C19—C20	1.372 (5)	Mo5—O17	1.955 (3)
C19—H19	0.9300	Mo5—O19	2.314 (2)
C20—N4	1.338 (4)	Mo6—O6	1.675 (3)
C20—H20	0.9300	Mo6—O12	1.887 (2)
C21—N6	1.341 (4)	Mo6—O8	1.891 (2)
C21—C22	1.376 (5)	Mo6—O16	1.956 (2)
C21—H21	0.9300	Mo6—O14	1.966 (2)
C22—C23	1.368 (6)	Mo6—O19	2.315 (2)
C22—H22	0.9300	N4—Ni1	2.066 (3)
C23—C24	1.372 (6)	N3—Ni1	2.078 (3)
C23—H23	0.9300	N2—Ni1	2.082 (3)
C24—C25	1.378 (5)	N6—Ni1	2.095 (3)
C24—H24	0.9300	N1—Ni1	2.101 (3)
C25—N6	1.342 (4)	N5—Ni1	2.105 (3)
C25—C26	1.481 (5)		
			
N1—C1—C2	123.1 (4)	O2—Mo3—O10	103.34 (12)
N1—C1—H1	118.5	O2—Mo3—O7	103.09 (13)
C2—C1—H1	118.5	O10—Mo3—O7	88.85 (11)
C1—C2—C3	118.3 (4)	O2—Mo3—O9	103.01 (12)
C1—C2—H2	120.8	O10—Mo3—O9	88.04 (10)
C3—C2—H2	120.8	O7—Mo3—O9	153.73 (10)
C4—C3—C2	119.2 (4)	O2—Mo3—O8	102.91 (12)
C4—C3—H3	120.4	O10—Mo3—O8	153.73 (10)
C2—C3—H3	120.4	O7—Mo3—O8	86.47 (11)
C3—C4—C5	119.6 (4)	O9—Mo3—O8	84.87 (10)
C3—C4—H4	120.2	O2—Mo3—O19	179.07 (11)
C5—C4—H4	120.2	O10—Mo3—O19	77.57 (8)
N1—C5—C4	121.0 (3)	O7—Mo3—O19	77.10 (9)
N1—C5—C6	116.4 (3)	O9—Mo3—O19	76.76 (9)
C4—C5—C6	122.6 (3)	O8—Mo3—O19	76.19 (8)
N2—C6—C7	121.1 (3)	O5—Mo4—O13	104.36 (12)
N2—C6—C5	115.0 (3)	O5—Mo4—O17	104.04 (12)
C7—C6—C5	123.8 (3)	O13—Mo4—O17	90.54 (11)
C8—C7—C6	119.6 (4)	O5—Mo4—O10	102.90 (12)
C8—C7—H7	120.2	O13—Mo4—O10	87.88 (11)
C6—C7—H7	120.2	O17—Mo4—O10	152.54 (10)
C9—C8—C7	119.2 (4)	O5—Mo4—O11	102.35 (12)
C9—C8—H8	120.4	O13—Mo4—O11	153.12 (10)
C7—C8—H8	120.4	O17—Mo4—O11	85.82 (11)
C10—C9—C8	118.9 (4)	O10—Mo4—O11	83.35 (10)
C10—C9—H9	120.5	O5—Mo4—O19	177.53 (11)
C8—C9—H9	120.5	O13—Mo4—O19	77.82 (9)
N2—C10—C9	123.2 (4)	O17—Mo4—O19	76.96 (9)
N2—C10—H10	118.4	O10—Mo4—O19	75.92 (8)
C9—C10—H10	118.4	O11—Mo4—O19	75.41 (9)
N3—C11—C12	123.1 (4)	O4—Mo5—O16	104.16 (13)
N3—C11—H11	118.4	O4—Mo5—O18	104.37 (14)
C12—C11—H11	118.4	O16—Mo5—O18	89.58 (11)
C13—C12—C11	118.7 (4)	O4—Mo5—O15	102.23 (14)
C13—C12—H12	120.7	O16—Mo5—O15	87.55 (11)
C11—C12—H12	120.7	O18—Mo5—O15	153.14 (10)
C12—C13—C14	119.2 (4)	O4—Mo5—O17	102.54 (13)
C12—C13—H13	120.4	O16—Mo5—O17	153.20 (10)
C14—C13—H13	120.4	O18—Mo5—O17	85.97 (11)
C15—C14—C13	119.4 (4)	O15—Mo5—O17	84.68 (11)
C15—C14—H14	120.3	O4—Mo5—O19	177.68 (13)
C13—C14—H14	120.3	O16—Mo5—O19	77.11 (9)
N3—C15—C14	121.8 (3)	O18—Mo5—O19	77.50 (9)
N3—C15—C16	114.7 (3)	O15—Mo5—O19	75.82 (9)
C14—C15—C16	123.5 (3)	O17—Mo5—O19	76.12 (9)
N4—C16—C17	121.3 (3)	O6—Mo6—O12	103.46 (12)
N4—C16—C15	115.7 (3)	O6—Mo6—O8	103.67 (12)
C17—C16—C15	123.0 (3)	O12—Mo6—O8	90.08 (10)
C18—C17—C16	118.9 (4)	O6—Mo6—O16	103.22 (12)
C18—C17—H17	120.5	O12—Mo6—O16	86.98 (11)
C16—C17—H17	120.5	O8—Mo6—O16	152.90 (10)
C19—C18—C17	119.4 (3)	O6—Mo6—O14	102.92 (12)
C19—C18—H18	120.3	O12—Mo6—O14	153.44 (10)
C17—C18—H18	120.3	O8—Mo6—O14	86.64 (11)
C18—C19—C20	118.3 (3)	O16—Mo6—O14	84.12 (11)
C18—C19—H19	120.9	O6—Mo6—O19	178.63 (11)
C20—C19—H19	120.9	O12—Mo6—O19	77.51 (9)
N4—C20—C19	123.2 (3)	O8—Mo6—O19	77.24 (9)
N4—C20—H20	118.4	O16—Mo6—O19	75.83 (9)
C19—C20—H20	118.4	O14—Mo6—O19	76.06 (9)
N6—C21—C22	122.2 (4)	C1—N1—C5	118.7 (3)
N6—C21—H21	118.9	C1—N1—Ni1	126.9 (2)
C22—C21—H21	118.9	C5—N1—Ni1	114.3 (2)
C23—C22—C21	119.0 (4)	C10—N2—C6	118.0 (3)
C23—C22—H22	120.5	C10—N2—Ni1	126.5 (2)
C21—C22—H22	120.5	C6—N2—Ni1	114.5 (2)
C22—C23—C24	118.7 (4)	C11—N3—C15	117.8 (3)
C22—C23—H23	120.7	C11—N3—Ni1	127.0 (2)
C24—C23—H23	120.7	C15—N3—Ni1	114.6 (2)
C23—C24—C25	120.5 (4)	C20—N4—C16	118.8 (3)
C23—C24—H24	119.8	C20—N4—Ni1	125.7 (2)
C25—C24—H24	119.8	C16—N4—Ni1	115.1 (2)
N6—C25—C24	120.5 (4)	C30—N5—C26	118.3 (3)
N6—C25—C26	115.6 (3)	C30—N5—Ni1	125.7 (2)
C24—C25—C26	123.9 (3)	C26—N5—Ni1	114.9 (2)
N5—C26—C27	121.7 (3)	C21—N6—C25	119.2 (3)
N5—C26—C25	115.2 (3)	C21—N6—Ni1	125.4 (3)
C27—C26—C25	123.0 (3)	C25—N6—Ni1	115.5 (2)
C28—C27—C26	119.0 (4)	N4—Ni1—N3	78.88 (11)
C28—C27—H27	120.5	N4—Ni1—N2	171.03 (11)
C26—C27—H27	120.5	N3—Ni1—N2	98.46 (11)
C29—C28—C27	119.9 (4)	N4—Ni1—N6	92.97 (11)
C29—C28—H28	120.0	N3—Ni1—N6	96.64 (11)
C27—C28—H28	120.1	N2—Ni1—N6	95.86 (11)
C28—C29—C30	118.5 (4)	N4—Ni1—N1	92.93 (11)
C28—C29—H29	120.7	N3—Ni1—N1	89.33 (10)
C30—C29—H29	120.7	N2—Ni1—N1	78.42 (11)
N5—C30—C29	122.5 (4)	N6—Ni1—N1	172.31 (11)
N5—C30—H30	118.7	N4—Ni1—N5	97.11 (11)
C29—C30—H30	118.7	N3—Ni1—N5	173.07 (11)
O1—Mo1—O11	103.71 (12)	N2—Ni1—N5	86.35 (11)
O1—Mo1—O9	104.51 (12)	N6—Ni1—N5	77.82 (11)
O11—Mo1—O9	89.01 (10)	N1—Ni1—N5	96.56 (11)
O1—Mo1—O18	102.16 (13)	Mo3—O7—Mo2	116.45 (12)
O11—Mo1—O18	87.81 (11)	Mo6—O8—Mo3	116.48 (11)
O9—Mo1—O18	153.14 (10)	Mo1—O9—Mo3	116.57 (11)
O1—Mo1—O12	103.36 (12)	Mo3—O10—Mo4	116.47 (12)
O11—Mo1—O12	152.83 (10)	Mo1—O11—Mo4	117.16 (12)
O9—Mo1—O12	86.40 (10)	Mo6—O12—Mo1	116.61 (11)
O18—Mo1—O12	84.37 (11)	Mo4—O13—Mo2	116.26 (12)
O1—Mo1—O19	178.41 (12)	Mo2—O14—Mo6	116.55 (12)
O11—Mo1—O19	77.08 (9)	Mo2—O15—Mo5	116.95 (12)
O9—Mo1—O19	76.84 (9)	Mo5—O16—Mo6	116.83 (12)
O18—Mo1—O19	76.45 (9)	Mo4—O17—Mo5	116.94 (12)
O12—Mo1—O19	75.79 (8)	Mo5—O18—Mo1	116.14 (12)
O3—Mo2—O14	104.40 (13)	Mo3—O19—Mo5	179.53 (11)
O3—Mo2—O15	103.63 (13)	Mo3—O19—Mo6	90.08 (7)
O14—Mo2—O15	90.04 (12)	Mo5—O19—Mo6	90.07 (7)
O3—Mo2—O7	103.27 (13)	Mo3—O19—Mo2	90.06 (7)
O14—Mo2—O7	87.90 (11)	Mo5—O19—Mo2	90.38 (7)
O15—Mo2—O7	152.67 (10)	Mo6—O19—Mo2	89.97 (7)
O3—Mo2—O13	102.07 (12)	Mo3—O19—Mo1	89.65 (7)
O14—Mo2—O13	153.52 (10)	Mo5—O19—Mo1	89.91 (7)
O15—Mo2—O13	84.70 (11)	Mo6—O19—Mo1	89.95 (7)
O7—Mo2—O13	85.10 (11)	Mo2—O19—Mo1	179.69 (11)
O3—Mo2—O19	178.16 (11)	Mo3—O19—Mo4	89.93 (7)
O14—Mo2—O19	77.42 (9)	Mo5—O19—Mo4	89.92 (7)
O15—Mo2—O19	76.57 (9)	Mo6—O19—Mo4	179.72 (12)
O7—Mo2—O19	76.39 (8)	Mo2—O19—Mo4	89.75 (7)
O13—Mo2—O19	76.12 (9)	Mo1—O19—Mo4	90.33 (7)

### Hydrogen-bond geometry (Å, °)

**Table:**

*D*—H···*A*	*D*—H	H···*A*	*D*···*A*	*D*—H···*A*
C29—H29···O11^i^	0.93	2.48	3.293 (5)	146.
C2—H2···O1^ii^	0.93	2.66	3.381 (5)	134.
C14—H14···O5^ii^	0.93	2.67	3.386 (4)	135.
C14—H14···O11^ii^	0.93	2.75	3.396 (4)	127.
C8—H8···O2^iii^	0.93	2.66	3.228 (5)	120.
C7—H7···O2^iii^	0.93	2.53	3.169 (4)	126.
C12—H12···O16^iv^	0.93	2.54	3.241 (4)	132.
C9—H9···O15^v^	0.93	2.36	3.165 (4)	144.
C21—H21···O3^vi^	0.93	2.54	3.163 (5)	124.

Symmetry codes: (i) −*x*+1/2, *y*+1/2, −*z*+3/2; (ii) −*x*+1, −*y*+1, −*z*+2; (iii) *x*−1/2, −*y*+1/2, *z*−1/2; (iv) −*x*+3/2, *y*+1/2, −*z*+3/2; (v) −*x*+1, −*y*+1, −*z*+1; (vi) *x*, *y*+1, *z*.
